# Association of On-Admission Anemia With 1-Year Mortality in Patients Hospitalized With Acute Heart Failure: Results From the HERO Study

**DOI:** 10.3389/fcvm.2022.856246

**Published:** 2022-05-04

**Authors:** Junlei Li, Chao Jiang, Yiwei Lai, Li Li, Xiaoyan Zhao, Xiaofang Wang, Ling Li, Xin Du, Changsheng Ma, Jianzeng Dong

**Affiliations:** ^1^Department of Cardiology, Beijing AnZhen Hospital, National Clinical Research Centre for Cardiovascular Diseases, Beijing Advanced Innovation Center for Big Data-Based Precision Medicine for Cardiovascular Diseases, Capital Medical University, Beijing, China; ^2^Department of Cardiology, The First Affiliated Hospital of Zhengzhou University, Zhengzhou, China; ^3^Heart Health Research Center (HHRC), Beijing, China; ^4^The George Institute for Global Health, Faculty of Medicine, University of New South Wales, Sydney, NSW, Australia

**Keywords:** acute heart failure (AHF), anemia, natriuretic peptides, volume overload, heart failure hospitalization

## Abstract

**Background:**

Anemia is common in patients with chronic heart failure (HF) and is associated with adverse outcomes. However, data regarding the prognostic value of on-admission anemia on mortality in patients hospitalized with acute HF were relatively limited and conflicting. This study aimed to investigate the association of on-admission anemia with 1-year mortality and evaluate whether anemia is an independent predictor of mortality in patients hospitalized with acute HF.

**Methods:**

The present analysis included 4,244 patients hospitalized with acute HF from the HERO (Heart Failure Registry of Patient Outcomes) study. On-admission anemia was defined using the World Health Organization (WHO) criteria (hemoglobin <120 g/L in women and <130 g/L in men). Cox proportional hazards models were used to assess the association of anemia with 1-year all-cause and cardiovascular mortality.

**Results:**

Of 4,244 patients, 2,206 (52.0%) patients had no anemia, 1,106 (26.1%) patients had mild anemia (men 110 ≤ hemoglobin < 130 g/L; women 110 ≤ hemoglobin < 120g/L), and 932 (22.0%) patients had moderate-to-severe anemia (hemoglobin < 110 g/L). After a median follow-up of 12.4 months (interquartile range: 11.9, 12.6), 867 (20.4%) patients died. Among the 742 (85.6%) deaths with confirmed causes, 664 (89.5%) were due to cardiovascular diseases. The mortality rates in patients with no anemia, mild anemia, and moderate-to-severe anemia were 16.6%, 20.4%, and 29.4%, respectively (*p* < 0.001). The association of anemia with increased all-cause mortality was significant in the unadjusted model (hazard ratio [HR]: 1.54, 95% confidential interval [CI]: 1.35–1.77, *p* < 0.001), and remained statistically significant after adjustment for most potential confounders (HR: 1.20, 95%CI: 1.03–1.40, *p* = 0.020), but no longer significant after additional adjustment for natriuretic peptides (HR: 1.02, 95%CI: 0.86–1.21, *p* = 0.843). When considering the degree of anemia, moderate-to-severe anemia was an independent predictor of all-cause mortality after full adjustment (HR:1.26, 95%CI: 1.03–1.54, *p* = 0.028), whereas mild anemia was not (HR: 0.84, 95%CI: 0.69–1.04, *p* = 0.104). A similar relationship was also found between anemia and cardiovascular mortality.

**Conclusions:**

On-admission anemia, defined by the WHO criteria, is not an independent predictor of mortality in patients hospitalized with acute HF. Moderate-to-severe anemia in patients with acute HF is independently associated with increased mortality.

## Introduction

Anemia is a highly prevalent comorbidity in patients with chronic heart failure (HF) and is associated with an increased risk of adverse outcomes (1). The reported prevalences of anemia in patients with HF were widely varying among different studies, ranging from 10% to 70%, mainly depending on the severity of HF and the definition of anemia (1–3). The etiologies of anemia in HF patients are multifactorial and heterogeneous, including inadequate erythropoietin levels due to renal dysfunction, hemodilution, hematinic deficiencies such as iron deficiency, bone marrow dysfunction, inflammation, and medications (4). Compared with chronic HF, progressive volume overload was more common in patients hospitalized with acute HF, which indicated that hemodilution might play a relatively larger role in anemia (5). Approximately half of the patients with acute HF had anemia (6); however, available data regarding the prognostic value of anemia in acute HF were relatively limited and conflicting. Amicis et al. and Rodríguez et al. reported that anemia was an independent predictor of mortality in patients with acute HF (7, 8). In contrast, Tymińska et al. found that the association of anemia with increased risk of mortality was significant in univariate analysis but no longer significant after adjustment for potential confounders (9). In the present study, we sought to assess the association of on-admission anemia with 1-year mortality in patients hospitalized with acute HF and evaluate whether anemia was an independent predictor of mortality in the acute setting of HF.

## Methods

### Study Population and Definitions

The study was performed in accordance with the principles of the Declaration of Helsinki. It was approved centrally by the Ethics Committee on Scientific Research and Clinical Trials at the First Affiliated Hospital of Zhengzhou University (in September 2017; approval number 2014SY-079) and by the local health research ethics board at each participating hospital. All patients gave written informed consent.

The Heart Failure Registry of Patient Outcomes (HERO) study is a prospective, multi-center, hospital-based cohort study designed to describe profile, management, and 1-year outcomes of patients hospitalized with acute HF in China (10). The HERO study consecutively recruited adult (≥18 years) patients from 73 participating hospitals with a primary admission diagnosis of acute HF during the defined period as previously described. The diagnosis of acute HF was made according to the 2016 European Society of Cardiology HF guidelines (11). From 10 November 2017 to 4 November 2018, 5,620 patients hospitalized with acute HF were enrolled into the HERO study. The socio-demographic characteristics, lifestyles, and self-reported health status were collected by physicians. The comorbidities, clinical characteristics at admission, laboratory tests, New York Heart Association (NYHA) class, treatments, and in-hospital outcomes were obtained from the medical records. According to the World Health Organization (WHO) criteria (12), patients were divided into three groups: non-anemia (hemoglobin ≥ 120 g/L in women or ≥130 g/L in men), mild anemia (110 ≤ hemoglobin <130g/L in men or 110 ≤ hemoglobin <120 g/L in women), and moderate-to-severe anemia (hemoglobin < 110 g/L).

### Follow-Up and Clinical Outcomes

In the HERO study, only patients discharged alive with consent to follow-up calls were enrolled in the prospective cohort. Standardized follow-up calls conducted by trained nurses were scheduled at 2 weeks, and 3, 6, and 12 months after discharge, or until death or study withdrawal. The outcomes of interest in the present analysis were 1-year all-cause and cardiovascular mortality.

### Statistical Analysis

Categorical variables were demonstrated as frequencies and percentages. The Chi-Square test was used for categorical variables comparison. Continuous variables were presented as mean ± standard deviation or median and interquartile range and compared with one-way analysis of variance (ANOVA) tests or Kruskal–Wallis tests as appropriate. A two-sided *p* < 0.05 was defined as statistical significance. Kaplan–Meier curves were plotted and compared using the log-rank test. Cox proportional hazards regression was used to evaluate the associations between anemia and all-cause and cardiovascular mortality in each group. Potential confounding variables were adjusted based on univariable regression (*P* < 0.1) and clinical knowledge. The adjusted variables in the Cox model included age, sex, body mass index (BMI), systolic blood pressure, current smoking, coronary artery disease, diabetes, chronic obstructive pulmonary disease (COPD), anemia, decreased eGFR (<60 mL/min/1.73 m^2^), hyponatremia (<135 mmol/L), in-hospital left ventricular ejection fraction groups (<40%, 40%−49%, ≥50%, or unavailable), and the use of renin-angiotensin system inhibitors, beta-blockers, mineralocorticoid receptor antagonists and statin at discharge, and hospital levels. Patients with missing covariate values were excluded, resulting in a total sample size of 3,568 patients for the adjusted analysis. Of 4,244 patients included in the present analysis, N-terminal pro-B-type natriuretic peptide (NT-proBNP) was measured in 2,360 patients during hospitalization. In patients without NT-proBNP, brain natriuretic peptide (BNP) was available in 1,103 patients. The values of BNP and NT-proBNP were log-transformed and standardized, respectively. Then, they were combined into a new variable, NPs, as described in a previous study (13) (Supplementary Materials). In the model-NPs, NPs values (presented as z score), as an indicator of HF severity and volume overload, were added to the adjusted model. A total of 2,928 patients with NPs values and without other missing covariate values were included in the model-NPs. In considering the possibility that the results of model-NPs were caused by patients' selection rather than the additional adjustment for NPs, we used multiple imputations to handle missing data in the sensitivity analysis. Multiple Cox regression analyses were performed to assess the effect of the additional adjustment for NPs on the main results. Potential nonlinear associations of hemoglobin levels with all-cause mortality were tested with restricted cubic splines. Knots were placed at the 25th, 50th, and 75th percentile of the distribution of hemoglobin values. Restricted cubic splines were performed with R (version 4.0.3). Other analyses were performed with IBM SPSS Statistics (version 26).

## Results

### Baseline Characteristics

**Figure 1:**
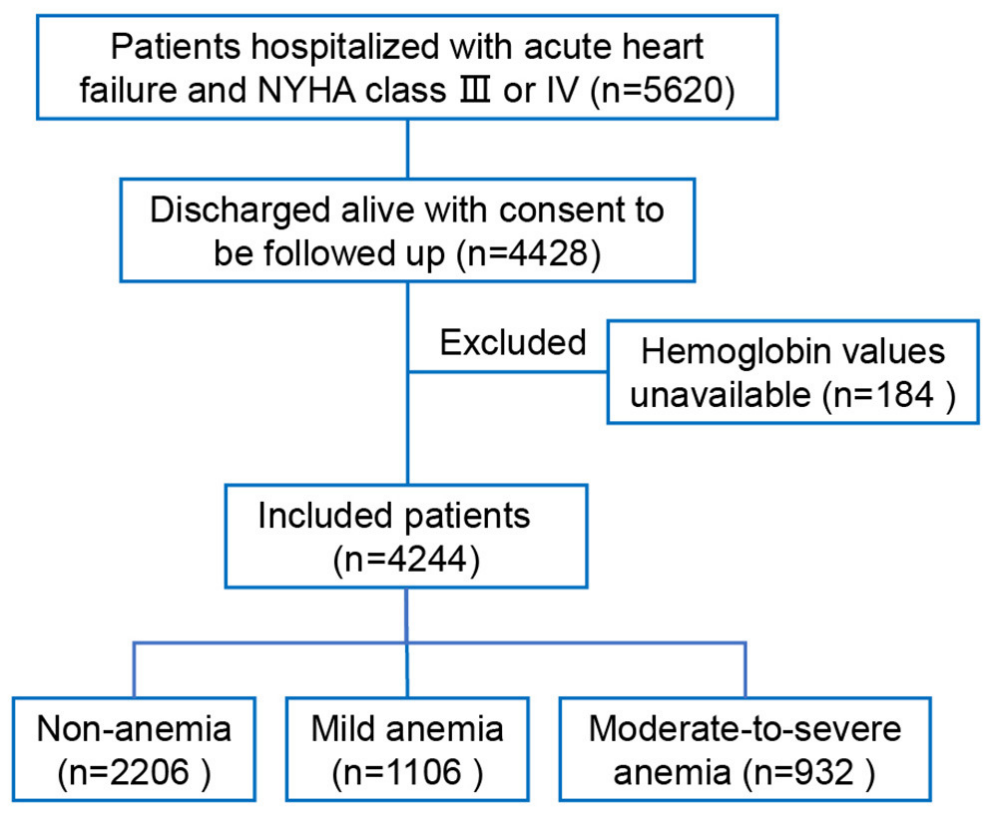
Flow chart.

Of 5,620 patients hospitalized with acute HF and NYHA class III or IV in the HERO study, 4,428 patients were discharged alive with consent to be followed up. After excluding 184 patients without available data on hemoglobin concentration, 4,244 patients were included in the present study (Figure 1). The rates of non-anemia, mild anemia, and moderate-to-severe anemia were 52.0%, 26.1%, and 22.0%, respectively. The median hemoglobin concentrations were 137 g/L (interquartile range [IQR]: 130, 147), 117 g/L (IQR: 114, 123), 99 g/L (IQR: 87, 105) in patients with non-anemia, mild anemia, and moderate-to-severe anemia, respectively. Table 1 shows the baseline characteristics according to the severity of anemia. In general, anemic patients were older, more likely to be female, and had lower BMI, worse HF severity, and more comorbidities. Anemic patients were less likely to receive Renin-angiotensin system inhibitors, β-blockers, and digoxin at discharge.

**Table 1:** Baseline characteristics.

**Variables**	**Non-anemia**	**Mild anemia**	**M-to-S anemia**	***P*-value**
Overall *n*, %	2,206 (52.0)	1,106 (26.1)	932 (22%)	-
**Socio-demographic**
Age, year, mea*n* (SD)	70 (12.1)	74 (11.2)	75 (12.0)	<0.001
Female sex, *n* (%)	1,052 (47.7)	438 (39.6)	602 (64.6)	<0.001
Low education, *n* (%)^a^	1,473 (76.0)	756 (79.2)	645 (78.8)	0.095
Low income, *n* (%)^b^	1,252 (66.7)	647 (69.4)	533 (67.3)	0.354
no/low insurance, *n* (%)^c^	1,652 (76.1)	859 (78.6)	699 (76.0)	0.240
Tertiary-level hospital, *n* (%)	537 (24.3)	229 (20.7)	192 (20.6)	0.016
Current smoking, *n* (%)	208 (9.5)	95 (8.6)	41 (4.4)	<0.001
Current drinking, *n* (%)	121 (5.5)	40 (3.6)	13 (1.4)	<0.001
**Clinical features**
Hemoglobin, g/L, mean (SD)	139 (12.7)	118 (5.6)	93 (17.1)	<0.001
BMI, kg/m2, mean (SD)	23.7 (3.9)	23.0 (3.6)	22.5 (5.4)	<0.001
SBP, mmHg, mean (SD)	135 (24.4)	134 (25.6)	136 (26.3)	0.160
Heart rate, mean (SD)	90 (22.9)	88 (22.7)	85 (22.0)	<0.001
LVEF, mean (SD)	49.2 (14.7)	49.0 (13.4)	50.1 (12.5)	0.337
NYHA class IV, *n* (%)	992 (45.0)	532 (48.1)	497 (53.3)	<0.001
BNP, pg/mL, median (IQR)^d^	836 (26,53,175)	1,093 (44,53,277)	1,484 (54,84,566)	<0.001
NT-proBNP, pg/mL, median (IQR)^e^	2,225 (70,85,960)	3,683 (12,708,561)	4,639 (1,85,011,486)	<0.001
eGFR <60mL/min/1.73m2, *n* (%)^f^	477 (22.9)	309 (29.7)	403 (46.3)	<0.001
Serum sodium, mean (SD)	139.4 (4.4)	138.7 (4.8)	138.1 (5.4)	<0.001
Serum potassium, mean (SD)	4.2 (0.6)	4.1 (0.6)	4.2 (0.8)	0.008
LDL-C, mmol/L, mean (SD)	2.29 (1.80, 2.93)	2.06 (1.61, 2.64)	2.00 (1.48, 2.60)	<0.001
**Medical history**
Hypertension, *n* (%)	1,036 (47.0)	503 (45.5)	486 (52.1)	0.026
Diabetes, *n* (%)	395 (18.0)	211 (19.2)	239 (25.7)	<0.001
Coronary artery disease, *n* (%)	611 (27.9)	369 (33.4)	269 (29.0)	0.005
COPD, *n* (%)	218 (9.9)	111 (10.1)	62 (6.7)	0.009
Atrial fibrillation, *n* (%)	597 (27.1)	298 (27.1)	220 (23.7)	0.112
Cerebrovascular disease, *n* (%)	311 (14.1)	174 (15.8)	145 (15.6)	0.355
valvular heart disease	530 (24.2)	263 (24.0)	217 (23.4)	0.882
Congenital heart disease, *n* (%)	29 (1.3)	11 (1.0)	11 (1.2)	0.871
**Treatment at discharge**
Renin-angiotensin system inhibitors, *n* (%)	1,018 (46.7)	483 (44.0)	374 (40.9)	0.010
β-blockers, *n* (%)	1,164 (53.2)	594 (53.9)	407 (44.4)	<0.001
MRA, *n* (%)	1,564 (71.4)	820 (74.4)	648 (70.7)	0.116
Diuretics, *n* (%)	1,304 (59.8)	668 (60.7)	539 (59.2)	0.781
Digoxin, *n* (%)	520 (23.8)	232 (21.0)	163 (17.8)	0.001
Statin, *n* (%)	1,511 (69.1)	759 (68.8)	597 (65.3)	0.103

*M-to-S anemia, moderate-to-severe anemia; Non-anemia (hemoglobin ≥ 120 g/L in women or ≥130 g/L in men); mild anemia (110 ≤hemoglobin <130 g/L in men or 110 ≤hemoglobin < 120 g/L in women); moderate-to-severe anemia (hemoglobin < 110 g/L). SD, standard deviation; IQR, interquartile range; SBP, Systolic blood pressure; BMI, body mass index; LVEF, left ventricular ejection fraction; NYHA, New York Heart Association; BNP, brain natriuretic peptide; NT-proBNP, N-terminal pro-B-type natriuretic peptide; eGFR, estimated glomerular filtration rate; LDL-C, low-density lipoprotein cholesterol; COPD, chronic obstructive pulmonary disease; MRA, mineralocorticoid receptor antagonists*.

a *Elementary school or below was defined as low education*;

b *Income < 30 k RMB per year was defined as low income*;

c *New rural cooperative medical scheme was defined as low-coverage insurance*;

d *On-admission NT-proBNP levels were available in 2,360 patients*.

e *On-admission BNP levels were available in 1,103 patients*.

f *eGFR was calculated with the Chronic Kidney Disease Epidemiology Collaboration (CKD-EPI) equation*.

### Clinical Outcomes

**Figure 2:**
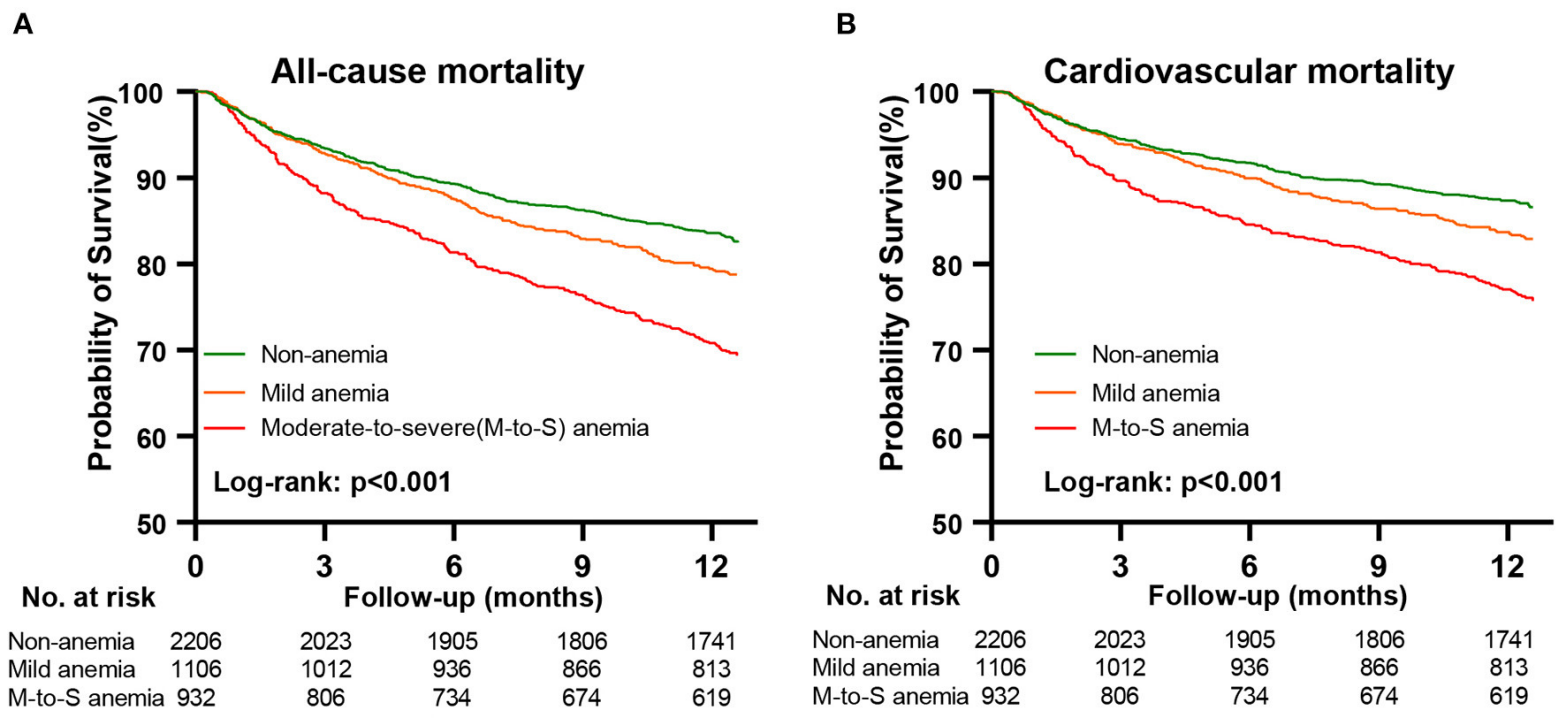
Kaplan-Meier curves for all-cause and cardiovascular mortality according to anemia. Panel **(A)**, Kaplan-Meier survival curves for all-cause mortality. Panel **(B)**, Kaplan-Meier survival curves for cardiovascular mortality. M-to-S anemia, moderate-to-severe anemia.

After a median follow-up of 12.4 months (IQR: 11.9, 12.6), 867 (20.4%) patients died. The causes of 867 deaths were cardiovascular in 664 (76.6%), non-cardiovascular in 78 (9.0%), and undetermined in 125 (14.4%). Kaplan-Meier curves for all-cause and cardiovascular mortality according to the severity of anemia are presented in Figure 2. The results of Cox regression analyses for all-cause and cardiovascular mortality are demonstrated in Table 2. Anemia was significantly associated with increased all-cause mortality in the unadjusted model (hazard ratio [HR]: 1.54, 95% confidential interval [CI]: 1.35–1.77, *p* < 0.001). Before NPs values were included in the adjusted model, the association of anemia with increased mortality remained statistically significant (HR: 1.20, 95% CI:1.03–1.40, *p* = 0.020) after adjustment for age, sex, BMI, systolic blood pressure, current smoker, diabetes, COPD, coronary heart disease, decreased eGFR, hyponatremia, in-hospital left ventricular ejection fraction, NYHA class and hospital levels, and the use of renin-angiotensin system inhibitors, β-blockers, mineralocorticoid receptor antagonists and statin at discharge. However, the association of anemia with all-cause mortality was no longer significant after additional adjustment for NPs (HR: 1.02, 95%CI: 0.86–1.21, *p* = 0.843). A similar relationship was also found between anemia and cardiovascular mortality. The HRs (95% CI) for cardiovascular mortality in unadjusted, adjusted, and NPs models were 1.58 (1.35–1.84, *p* < 0.001), 1.22 (1.02–1.45, *p* = 0.027), and 1.01 (0.84–1.23, *p* = 0.895). When considering the degree of anemia, moderate-to-severe anemia was an independent predictor of mortality after full adjustment (HR:1.26, 95%CI: 1.03–1.54, *p* = 0.028), whereas mild anemia was not (HR: 0.87, 95%CI: 0.69–1.04, *p* = 0.104). The association of moderate-to-severe anemia with cardiovascular mortality showed a similar trend but did not reach statistical difference after full adjustment (HR:1.20, 95%CI: 0.96–1.51, *p* = 0.117, Table 2). In the sensitivity analysis, which used multiple imputations to handle the missing data, the findings that the association of anemia with all-cause and cardiovascular mortality remained significant after adjustment for most potential confounding factors but no longer significant after additional adjustment for NPs values were consistent (Supplementary Materials). Figure 3 demonstrated the HR (95%CI) for all-cause mortality according to hemoglobin levels in unadjusted, adjusted, and NPs models.

**Table 2:** The association of anemia with all-cause and cardiovascular mortality.

	**No. of events**	**Unadjusted model**		Adjusted model^*^		**Model-NPs** ^#^	
		**HR (95%CI)**	***P*-value**	**HR (95%CI)**	***P*-value**	**HR (95%CI)**	***P*-value**
**All-cause mortality**
Non-anemia	367/2,206 (16.6%)	1.00 (reference)	-	1.00 (reference)	-	1.00 (reference)	-
Any anemia	500/2,038 (24.5%)	1.54 (1.35–1.77)	<0.001	1.20 (1.03–1.40)	0.020	1.02 (0.86–1.21)	0.843
Mild anemia	226/1,106 (20.4%)	1.25 (1.06–1.48)	0.008	0.99 (0.82–1.19)	0.874	0.84 (0.69–1.04)	0.104
M-to-S anemia	274/932 (29.4%)	1.91 (1.63–2.23)	<0.001	1.50 (1.25–1.80)	<0.001	1.26 (1.03–1.54)	0.028
**Cardiovascular mortality**
Non-anemia	277/2,206 (12.6%)	1.00 (reference)	-	1.00 (reference)	-	1.00 (reference)	-
Any anemia	387/2,038 (19.0%)	1.58 (1.35–1.84)	<0.001	1.22 (1.02–1.45)	0.027	1.01 (0.84–1.23)	0.895
Mild anemia	177/1,106 (16.0%)	1.30 (1.08–1.57)	0.007	1.05 (0.85–1.29)	0.650	0.87 (0.71–1.13)	0.345
M-to-S anemia	210/932 (22.5%)	1.93 (1.61–2.31)	<0.001	1.44 (1.17–1.77)	0.001	1.20 (0.96–1.51)	0.117

*M-to-S anemia, moderate-to-severe anemia; Non-anemia (hemoglobin ≥ 120 g/L in women or ≥ 130 g/L in men); mild anemia (110 ≤ hemoglobin <130 g/L in men or 110 ≤ hemoglobin <120 g/L in women); moderate-to-severe anemia (hemoglobin <110 g/L)*.

* *Adjusted variables: age, sex, body mass index, systolic blood pressure, current smoker, diabetes, chronic obstructive pulmonary disease, coronary heart disease, decreased estimated glomerular filtration rate, hyponatremia, in-hospital left ventricular ejection fraction, New York Heart Association class, and the use of renin-angiotensin system inhibitors, β-blockers, mineralocorticoid receptor antagonists and statin at discharge, and hospital levels*.

# *Adjusted variables in Model-NPs: all variables mentioned above and additional adjustment for log-transformed and standardized natriuretic peptides levels. HF, heart failure; HR, hazard ratio; CI: confidence interval*.

**Figure 3:**
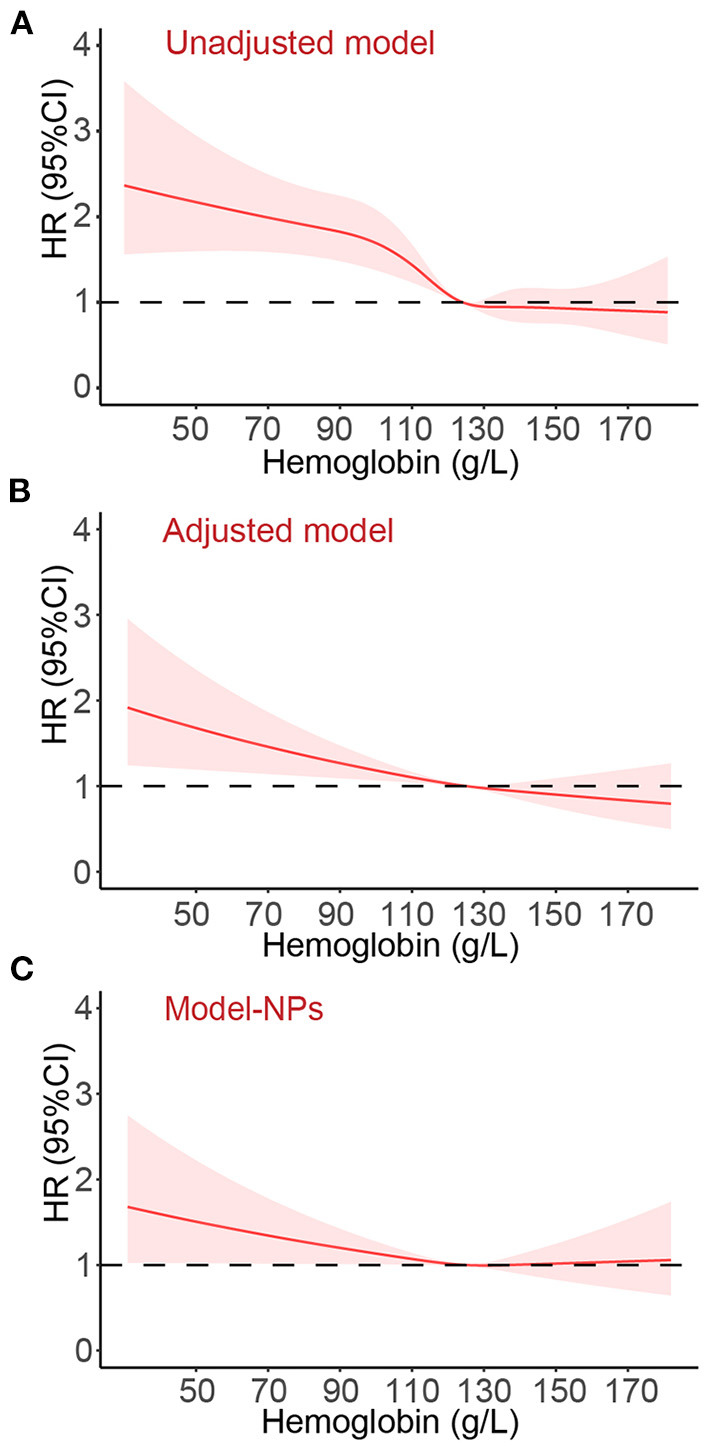
The hazard ratio (95% confidence interval) for all-cause death according to the hemoglobin levels. Panel **(A)**, unadjusted model. Panel **(B)**, Adjusted variables: age, sex, body mass index, systolic blood pressure, current smoker, diabetes, chronic obstructive pulmonary disease, coronary heart disease, decreased estimated glomerular filtration rate, hyponatremia, in-hospital left ventricular ejection fraction, New York Heart Association class, and the use of renin-angiotensin system inhibitors, β-blockers, mineralocorticoid receptor antagonists and statin at discharge, and hospital levels. Panel **(C)**, Adjusted variables in Model-NPs: all variables mentioned above and additional adjustment for log-transformed and standardized natriuretic peptides levels. HR, hazard ratio; CI, confidence interval.

## Discussion

In this large-scale prospective study of patients hospitalized with acute HF, on-admission anemia was present in almost half of the patients. The association of anemia with increased 1-year all-cause and cardiovascular mortality was significant in the unadjusted model and attenuated after adjustment for most potential confounders but remained statistically significant. Only after additional adjustment for natriuretic peptides, on-admission anemia was no longer a significant predictor of all-cause or cardiovascular mortality. When considering the degree of anemia, moderate-to-severe anemia was an independent predictor of mortality after full adjustment, whereas mild anemia was not.

Most previous studies regarding the prognostic value of anemia in HF were conducted in patients with chronic HF (1, 14). A large-scale meta-analysis including 1,53,180 patients with chronic HF demonstrated that the crude mortality rate of anemic patients was twice that of non-anemic patients, and this association remained significant after adjustment (adjusted HR: 1.46, 95%CI: 1.26–1.69) (1). The anemic rate tends to be higher in patients with acute HF than in patients with chronic HF; however, the impact of anemia on mortality in the acute setting is controversial (7–9). In the present study, the anemic patients with acute HF had a 54% increase in the risk of all-cause mortality and a 58% increase in the risk of cardiovascular mortality before the adjustment; however, after the additional adjustment for natriuretic peptides, the association was no longer significant. Interestingly, the two previous studies (7, 8), which concluded that anemia was an independent predictor of mortality, did not include natriuretic peptides values in the multivariate Cox model.

The discrepancies in the prognostic value of anemia between acute and chronic HF may partly be due to the differences in the etiology of anemia (4). Several small studies in patients with chronic HF have established the role of hemodilution caused by volume overload in anemia. Androne et al. measured the red blood cell and plasma volume in 37 anemic patients with chronic HF using a radiolabeled albumin technique and found that anemia in 17 patients was due to hemodilution, and only 20 patients had true anemia (15). Previous studies also demonstrated that the increases in plasma volume and extracellular volume were independent predictors of low hemoglobin levels in patients with chronic HF (16, 17). Progressive volume overload and congestion are the main reasons for hospital admission in patients with acute HF (18). Compared to the chronic setting, greater volume expansion and hemodilution during HF decompensation may be partly responsible for the higher anemic rate in patients hospitalized with acute HF (16), thus leading to a relatively higher proportion of pseudo-anemia. This might be the reason why natriuretic peptides, the markers of elevated filling pressure and volume overload (19), were the critical confounding factor that influenced the impact of anemia on all-cause and cardiovascular mortality in the present study. This is to say that hemoglobin levels are probably more like a marker of volume overload in the acute setting of HF. Diuretics are the cornerstone of acute HF treatment (20). Some pseudo-anemia caused by transient hemodilution might be corrected after effective hemoconcentration during hospitalization (15). Meanwhile, effective hemoconcentration during acute HF hospitalization is a predictor of improved prognosis (21, 22) and thus may mask the impact of anemia on mortality. However, there were no available data on the dynamic changes of hemoglobin levels before and after discharge in the HERO study; therefore, further studies are needed to clarify the detailed association of anemia with hemodilution and hemoconcentration in the acute setting of HF.

In addition, anemic patients were older and were more likely to have impaired renal function, lower BMI, diabetes, hypertension, and higher NYHA class. In the present study, the rate of impaired renal function (eGFR <60 mL/min/1.73 m^2^) in patients with moderate-to-severe anemia was about twice that of those without anemia and about 1.5 folds that of those with mild anemia. The significant association of anemia with adverse outcomes in unadjusted model and in previous studies may also be the results secondary to a higher comorbidity burden. Our study found that the usage rates of renin-angiotensin system inhibitors and β-blockers in anemic patients were significantly lower than those without anemia. This may be explained by possible intolerance of the medications due to worse HF severity or more renal dysfunction in anemic patients.

The present study showed that moderate-to-severe anemia was an independent predictor of all-cause mortality. This may be because a higher proportion of patients with moderate-to-severe anemia have real anemia, and anemia in these patients may still exist even after effective hemoconcentration. From this perspective, the cut-off point of WHO criteria may be relatively high to define anemia in patients with acute HF. Whether anemia in HF was a treatable target or merely a marker of HF severity or other comorbidities had been debated (4). In patients with chronic HF and anemia, extraneous erythropoietin did increase the hemoglobin concentration but failed to improve the prognosis of HF (23). However, a meta-analysis of randomized controlled trials demonstrated that intravenous iron therapy might improve the symptoms and the prognosis of systolic HF (24). Our findings suggest that mild anemia in acute HF may be more a marker of volume overload, whereas moderate-to-severe anemia should draw the attention of clinicians. Further studies are needed to explore whether specific treatment such as intravenous iron therapy will improve prognosis in patients hospitalized with acute HF and moderate-to-severe anemia.

### Limitations

First, fluid volume overload and hemodilution were common in patients hospitalized with acute HF. Some patients may experience effective diuresis and hemoconcentration after hospitalization. However, there were only baseline data of hemoglobin in our study. Second, the etiology of anemia in patients with HF is multifactorial, whereas there was no data on hematinics such as iron, Vitamin B12, and folate in the present study. Third, there was no information regarding the treatment of anemia.

## Conclusion

On-admission anemia, defined by the WHO criteria, is not an independent predictor of mortality in patients hospitalized with acute HF. Moderate-to-severe anemia in patients with acute HF is independently associated with increased mortality.

## Data Availability Statement

The original contributions presented in the study are included in the article/Supplementary Material, further inquiries can be directed to the corresponding authors.

## Ethics Statement

The studies involving human participants were reviewed and approved by The First Affiliated Hospital of Zhengzhou University. The patients/participants provided their written informed consent to participate in this study. Written informed consent was obtained from the individual (s) for the publication of any potentially identifiable images or data included in this article.

## Author Contributions

JD, XD, and JL contributed to conception and design of the study. XW, XZ, LingL, and LiL participated in the collection of samples and data. JL and YL performed statistical analysis and all the results were checked by CJ and LiL. JL wrote the first draft of the manuscript. LiL, YL, XZ, XW, and CJ wrote sections of the manuscript. The manuscript was critically revised by CM, XD, and JD. All authors contributed to the article and approved the submitted version.

## Funding

The study has been funded by the National Thirteenth 5-year Science and Technology Support Projects by the Ministry of Science and Technology of China (Grant No. 2016YFC1301000).

## Conflict of Interest

The authors declare that the research was conducted in the absence of any commercial or financial relationships that could be construed as a potential conflict of interest.

## Publisher's Note

All claims expressed in this article are solely those of the authors and do not necessarily represent those of their affiliated organizations, or those of the publisher, the editors and the reviewers. Any product that may be evaluated in this article, or claim that may be made by its manufacturer, is not guaranteed or endorsed by the publisher.
