# Supplementary material for: Association of On-Admission Anemia With 1-Year Mortality in Patients Hospitalized With Acute Heart Failure: Results From the HERO Study
Source: Front Cardiovasc Med. 2022 May 4;9:856246. doi: 10.3389/fcvm.2022.856246 (PMC9114434; doi:10.3389/fcvm.2022.856246)
Supplement: Supplementary file 1 [file Data_Sheet_1.doc]

**Supplementary materials**

##### Details of the log transformation and standard normal transformation for natriuretic peptides (NPs).

1. NT-proBNP levels were measured in 2360 patients during hospitalization. The distribution of NT-proBNP values was skewed (median, 3030.00; IQR, 978.35, 7689.35).
2. After log transformation (the base was 10), the values of LgNT-proBNP were normally distributed (mean±SD, 3.3649±0.7024).
3. According to the formula for standard normal transformation [z score=(x-mean)/SD], where standard LgNT-proBNP= (LgNTproBNP- 3.3649)/ 0.7024. The mean(±SD) of standard LgNT-proBNP was 0.00(±1.00).
4. Of 1884 patients without NT-proBNP, BNP levels were measured in 1103 patients during the hospitalization. The processes of log transformation and standard normal transformation were similar to NT-proBNP. The mean(±SD) of standard LgBNP values in the 1103 patients was also 0.00(±1.00).
5. Subsequently, we combined the values of standard LgNT-proBNP and standard LgBNP as a new variable, NPs. The table below shows the median, IQR, mean and Std deviation during log transformation and standard normal transformation.

##### The association of anemia with all-cause and cardiovascular mortality (multiple imputations)

|  | **No. of events** | **Unadjusted model** | | **Adjusted model*** | | **Model-NPs#** | |
| --- | --- | --- | --- | --- | --- | --- | --- |
|  |  | **HR (95%CI)** | **P value** | **HR (95%CI)** | **P value** | **HR (95%CI)** | **P value** |
| **All-cause mortality** | | | | | | | |
| Non-anemia | 367/2206(16.6%) | 1.00 (reference) | - | 1.00 (reference) | - | 1.00 (reference) | - |
| Any anemia | 500/2038(24.5%) | 1.54(1.35-1.77) | <0.001 | 1.18(1.03-1.36) | 0.020 | 1.11(0.96-1.28) | 0.148 |
| Mild anemia | 226/1106(20.4%) | 1.25(1.06-1.48) | 0.008 | 0.99(0.83-1.17) | 0.886 | 0.94(0.79-1.11) | 0.460 |
| M-to-S anemia | 274/932(29.4%) | 1.91(1.63-2.23) | <0.001 | 1.43(1.21-1.69) | <0.001 | 1.33(1.13-1.57) | 0.001 |
| **Cardiovascular mortality** | | | | | | | |
| Non-anemia | 277/2206(12.6%) | 1.00 (reference) | - | 1.00 (reference) | - | 1.00 (reference) | - |
| Any anemia | 387/2038(19.0%) | 1.58(1.35-1.84) | <0.001 | 1.19(1.01-1.39) | 0.035 | 1.11(0.94-1.30) | 0.216 |
| Mild anemia | 177/1106(16.0%) | 1.30(1.08-1.57) | 0.007 | 1.02(0.84-1.24) | 0.854 | 0.96(0.79-1.17) | 0.684 |
| M-to-S anemia | 210/932(22.5%) | 1.93(1.61-2.31) | <0.001 | 1.40(1.16-1.69) | <0.001 | 1.29(1.07-1.56) | 0.009 |

M-to-S anemia, moderate-to-severe anemia; Non-anemia (hemoglobin≥120g/L in women or ≥130g/L in men); mild anemia (110≤hemoglobin<130g/L in men or 110≤hemoglobin <120g/L in women); moderate-to-severe anemia (hemoglobin<110g/L). *Adjusted variables: age, sex, body mass index, systolic blood pressure, current smoker, diabetes, chronic obstructive pulmonary disease, coronary heart disease, decreased estimated glomerular filtration rate, hyponatremia, in-hospital left ventricular ejection fraction, New York Heart Association class, and the use of renin-angiotensin system inhibitors, β-blockers, mineralocorticoid receptor antagonists and statin at discharge, and hospital levels. #Adjusted variables in Model-NPs: all aforementioned variables and additional adjustment for log-transformed and standardized natriuretic peptides levels. HF, heart failure; HR, hazard ratio; CI: confidence interval.
